# Resource Utilization of Peanut Shells: Nutritional Characteristics, Regulation of Antinutritional Factors, and Application Potential in Livestock and Poultry Production

**DOI:** 10.1002/fsn3.70994

**Published:** 2025-12-06

**Authors:** Mengen Zhang, Dian Wang, Shiqin Wang, Hongsheng Du, Guodong Li, Rubing Lan, Yingli Li

**Affiliations:** ^1^ Youran Dairy Co., Ltd. Hohhot China; ^2^ Anhui Province Key Laboratory of Animal Nutritional Regulation and Health, College of Animal Science Anhui Science and Technology University Chuzhou China

**Keywords:** application in livestock and poultry production, bioactive compounds, nutritional value, peanut shell, regulation of anti‐nutritional factors

## Abstract

In agricultural production, peanut shells, as a by‐product of *peanut* processing, are often discarded. However, they are rich in fiber, crude protein, carbohydrates, minerals (such as calcium and phosphorus), as well as bioactive substances like polyphenols and flavonoids, thus having potential feeding value. Currently, the application potential of peanut shells in the field of animal nutrition has not been fully explored. The lack of comprehensive and systematic studies on their nutritional composition, digestive and metabolic characteristics, and optimal addition levels in different animal production systems limits the accurate evaluation and scientific application of their feeding value. In addition, different processing methods have significantly different effects on improving the nutritional components of peanut shells, and they contain anti‐nutritional factors such as phytic acid and oxalic acid, whose contents vary greatly with *peanut* varieties and producing areas, further increasing the complexity of evaluating their feeding value. Although relevant studies have achieved certain results, there are still many issues in the development and utilization of peanut shells that need further in‐depth exploration and optimization. This review will comprehensively evaluate the nutritional value of peanut shells, analyze in detail their conventional nutritional components, bioactive substances, and anti‐nutritional factors, so as to clarify their true value in animal nutrition. Meanwhile, it will conduct in‐depth research on the application effects of peanut shells in different livestock feeding systems. Through these studies, it aims to provide a scientific basis for the reasonable and efficient application of peanut shells in livestock feeding and offer new ideas and methods for solving the current problems of feed resource shortage and environmental issues.

## Graphical abstract

The article reviews the potential of peanut hulls as livestock feed, highlighting their nutritional components, processing methods, and challenges. Despite being rich in fiber, minerals, and bioactive substances, peanut hulls contain anti‐nutritional factors. Processing techniques like grinding, alkali treatment, and fermentation can enhance their value. They are used in various animal productions to improve growth and the environment but face issues like high costs and regulatory limitations. Future research should focus on optimizing their use and addressing these challenges.

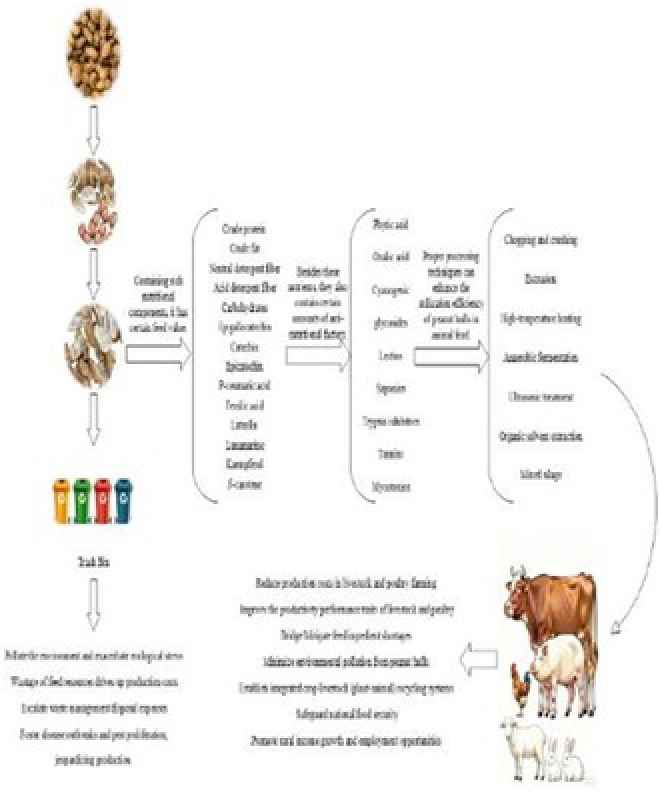

## 1 Introduction

**FIGURE 1:**
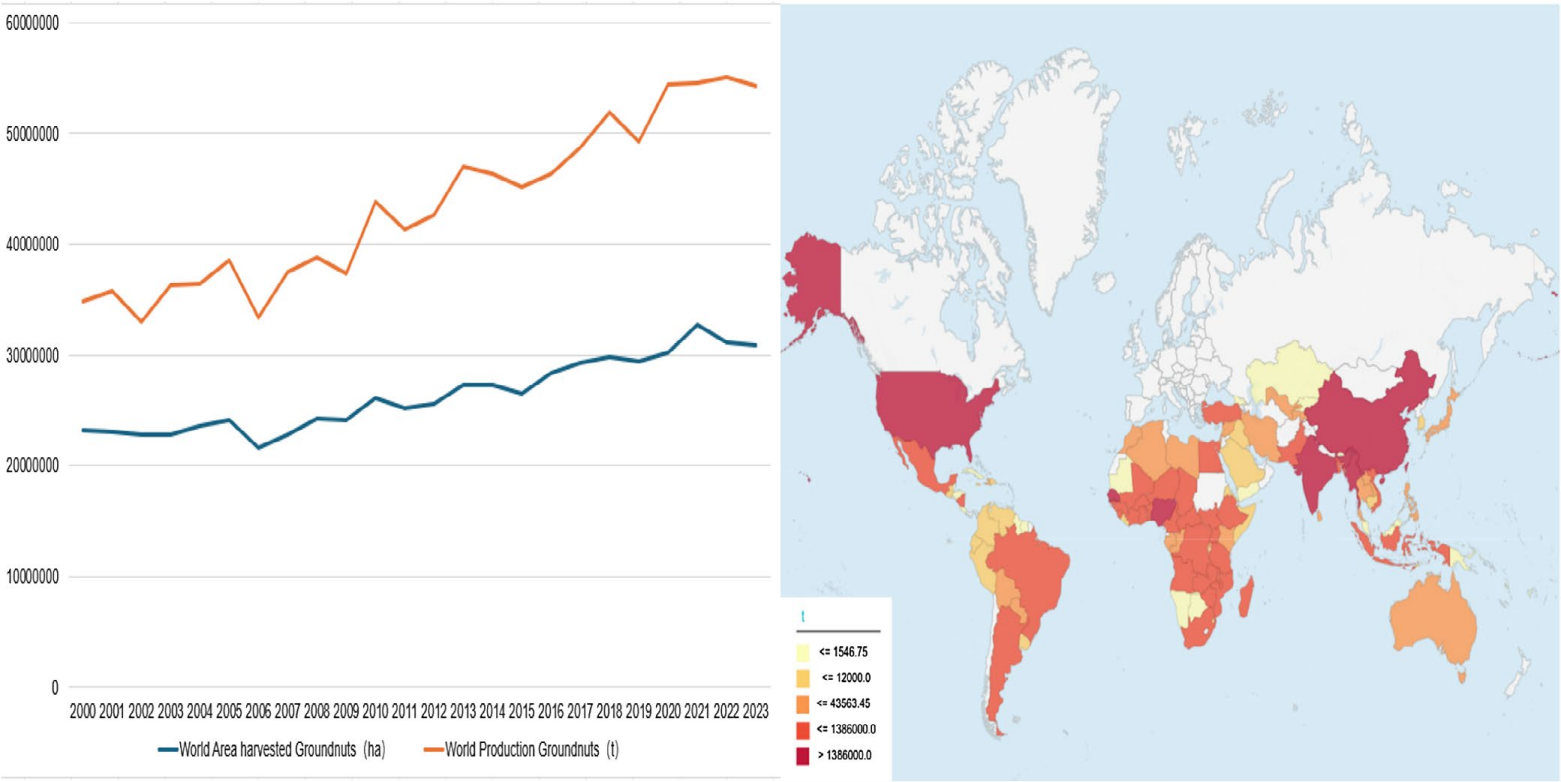
The global production/yield quantities of groundnuts, excluding shelled ones, from the year 2000–2023 (lift), along with the breakdown of production quantities by country in 2023 (right) (FAOSTAT 2023).

**FIGURE 2:**
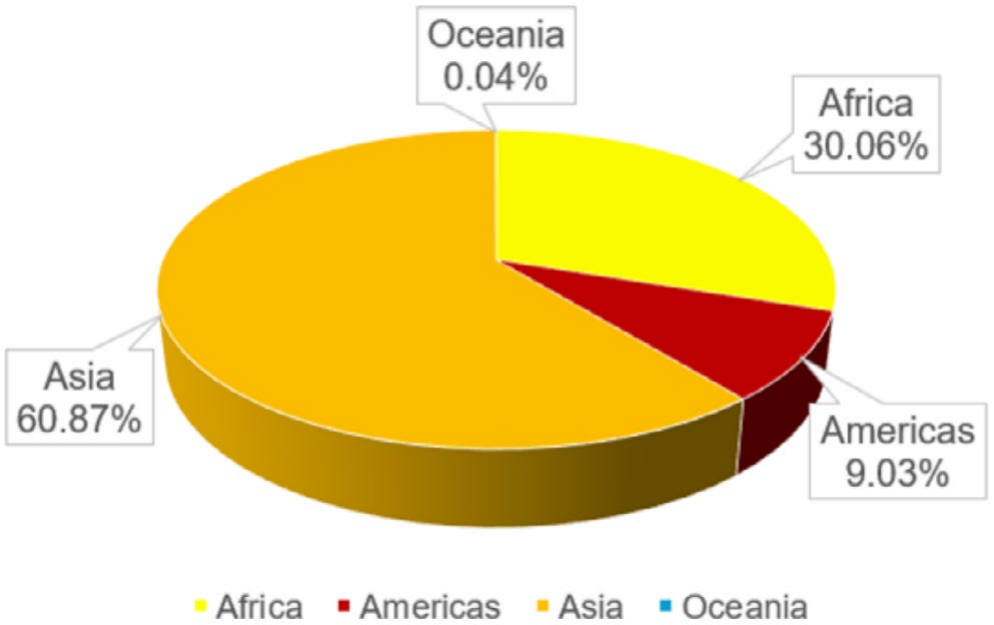
Production share of groundnuts, excluding shelled, by region 2023 (FAOSTAT 2023).

Peanut hulls, the primary by‐product of *Arachis hypogaea* L. processing, have historically been underexploited and dismissed as agricultural waste. As global peanut production has risen annually (both cultivation area and output exhibiting an upward trajectory from 2000 to 2023, Figure 1, left panel) (FAOSTAT 2023), peanut hull generation has correspondingly escalated. FAO statistics indicate global peanut production attained 54,272,900.42 metric tons in 2023, with Asia dominating global production (60.87%), followed by Africa (29.06%), the Americas (9.03%), and Oceania (0.04%) (Figure 1, right panel and Figure 2) (FAOSTAT 2023). Of the top five producers, China led with 19,230,700 tons, followed by India (10,296,708 tons), Nigeria (4,300,000 tons), the U.S. (2,671,670 tons), and Myanmar (1,795,642 tons). Using a conversion factor of 230–300 g hulls/kg peanuts (Zhao et al. 2012), global peanut hull output in 2023 is estimated at 12,482.77–16,281.87 metric tons. Vast quantities of peanut hulls remain underutilized. Improperly disposed or stockpiled hulls contaminate soils, degrading structural integrity and altering fertility dynamics. Rainfall‐induced leaching or irrigation runoff transports hulls into surface waters, triggering eutrophication and disrupting aquatic ecosystems. Spontaneous fermentation releases toxic gases, contributing to air pollution and posing fire risks. Their high bulk density and slow degradation rate necessitate extensive land use, hindering sustainable agricultural practices. Conventional disposal methods‐open burning and landfilling‐result in resource wastage while exacerbating environmental crises. Incineration releases particulate matter and greenhouse gases (e.g., CO_2_, NO), whereas landfills generate methane from lignocellulosic decomposition, intensifying the greenhouse effect (Bobet et al. 2020). Against this backdrop, developing sustainable valorization strategies for peanut hulls emerges as a critical priority in global agricultural waste governance.

Recently, peanut shells have garnered significant scientific interest due to their emerging potential value. Chemical analyses reveal a composition dominated by cellulose (48%), hemicellulose (3%), and lignin (28%) (Bobet et al. 2020), with notable contents of crude protein (4.8%–7.2%), essential minerals (Ca, P), and bioactive compounds including polyphenols and flavonoids (Huang et al. 2016; Adhikari et al. 2019). Notably, *luteolin*—a predominant flavonoid in peanut hulls—exerts antioxidant, anti‐inflammatory, and lipid metabolism‐modulating effects, contributing substantially to animal health improvement (Liu, Wang, and Zhang 2022). Nevertheless, several constraints impede direct utilization in animal feed: low soluble crude protein (30.62%–43.88%), elevated non‐protein nitrogen (56.13%–83.25%), minimal soluble true protein, excessive fiber content, poor digestibility, a high unusable cell wall proportion (47.94%), scarcity of non‐structural carbohydrates/pectin, and antinutritional factors like phytic and oxalic acids (Huang et al. 2016; Abdulrazak et al. 2014). Despite these challenges, their fibrous structure and nutrient profile position peanut hulls as a promising feed ingredient. Empirical evidence demonstrates that appropriately processed peanut hulls, when integrated into formulations, function as a cost‐effective, efficient, and eco‐friendly source of dietary fiber and energy (Mokolopi 2022). To overcome these limitations, diverse processing technologies have been devised to elevate the nutritional quality and bioavailability of peanut hulls. Physical interventions such as mechanical grinding effectively diminish fiber crystallinity, achieve a 26.65% reduction in crude fiber, and boost protein digestibility by 74.70% (Feng et al. 2022). Alkali (NaOH) treatment coupled with extrusion cooking engenders a linear decline in neutral detergent fiber and doubles in vitro digestibility relative to controls (Chandra et al. 1985). Microbial fermentation via probiotics markedly elevates soluble crude protein levels, suppresses pathogenic bacterial proliferation, and mitigates manure malodor (Pan et al. 2019).

Currently, peanut hulls are utilized across diverse livestock sectors‐poultry (Han et al. 2016; Armayanti et al. 2021), monogastric animals (Chao and Li 2008; Recharla et al. 2019; Zhang et al. 2022), and ruminants (Ciriaco et al. 2016; Yuan and Wan 2019; Mokolopi et al. 2021). Nevertheless, the nutritional potential of peanut hulls in animal diets remains underexplored. Limitations persist due to the scarcity of systematic investigations into their proximate composition, digestibility, metabolic fate, and optimal inclusion rates across production systems—factors critical for precise valuation and evidence‐based deployment in animal nutrition. Furthermore, processing efficacy exhibits substantial variability in nutrient enhancement, while inherent antinutrients (e.g., phytic and oxalic acids) compound evaluative challenges through cultivar‐ and origin‐dependent concentration fluctuations. This review rigorously appraises the nutritional profile of peanut hulls, dissecting conventional nutrients, bioactive constituents, and antinutritive factors to quantitatively define their functional role in animal metabolism. Concurrently, empirical effects across livestock feeding regimes are synthesized, encompassing growth metrics, feed intake, digestibility coefficients, health parameters, and output traits (meat quality, lactation efficiency, egg quality). Findings establish a scientific framework for optimized utilization of peanut hulls in rations, advancing sustainable solutions to feed resource scarcity and environmental stewardship challenges.

## 2 Nutritional Components and Value of Peanut Hulls

### 2.1 Conventional Nutritional Components of Peanut Hulls

In the field of unconventional feed resource utilization, peanut hulls, as a by‐product of peanut processing, have gradually attracted the attention of researchers. Their conventional nutrient composition is rich, showing significant potential for resource utilization. Existing studies have shown that the crude fiber content of peanut hulls ranges from 65.7% to 79.3%, with the fiber mainly consisting of cellulose (48%), hemicellulose (3%), and lignin (28%), followed by crude protein (4.8%–7.2%), crude fat (1.2%–1.8%), reducing sugars (0.3%–1.8%), disaccharides (1.7%–2.5%), pentoses (16.1%–17.8%), and starch (0.7%) (Yang et al. 2003). They also contain minerals such as calcium (0.20%), phosphorus (0.06%), magnesium (0.07%), potassium (0.57%), nitrogen (1.09%), iron (262 mg/kg), manganese (45 mg/kg), zinc (13 mg/kg), copper (10 mg/kg), boron (13 mg/kg), aluminum (454 mg/kg), strontium (262 mg/kg), barium (16 mg/kg), and sodium (66 mg/kg) (Yang et al. 2003). The nutrient composition determination results of peanut hulls vary slightly among different studies. The analysis by Imran et al. (2022) demonstrates that the moisture content, protein, fiber, fat, carbohydrate, ash, and nitrogen‐free extract contents of peanut hulls are 7.21%, 4.05%, 55.95%, 0.32%, 23.98%, 3.29%, and 5.01%, respectively. In the study by Varma et al. (2020), the following values were observed: moisture content (8.0%), ash (2.50%), crude fiber (59.0%), lipid (0.50%), and crude protein (4.43%). Collins and Post (1981) reported that two types of peanut hull powder had the highest concentrations of P, Ca, Mg, and K in the ash, with contents of 317–687.3, 362.1–401.3, 91.3–147, and 89.1–140.4 mg/100 g, respectively. From a nutritional structure perspective, peanut hulls are primarily composed of cellulose and hemicellulose, classifying them as typical high‐fiber and low‐fat raw materials. Liu et al. (1994) found that peanut hull cellulose is mainly composed of glucose and xylose, with relatively high glucose and galactose contents and low arabinose content, resulting in a hexose‐to‐pentose ratio of 3.4:1. Meanwhile, the polysaccharides in raw hull lignin are predominantly glucose, xylose, and mannose, with a hexose‐to‐pentose ratio of 2.1:1 (Liu et al. 1994). Among the five shell‐based feedstuffs (peanut husk, rice hull, sunflower seed hull, rapeseed pod hull, and cottonseed hull), peanut husk exhibits distinctive nutritional attributes and application potential (Table 1). It has the highest dry matter content, significantly surpassing other raw materials, which indicates minimal moisture levels, enhanced storage stability, and greater suitability for long‐term preservation and processing. Although its organic matter content is slightly lower than that of sunflower seed hull, it markedly exceeds that of rice hull, rapeseed pod hull, and cottonseed hull, reflecting a higher proportion of bioavailable carbon‐based nutrients that provide a superior foundation for livestock energy and nutrition. In terms of protein nutritional value, peanut husk is surpassed only by rapeseed pod hull in crude protein content and significantly outperforms rice hull, sunflower seed hull, and cottonseed hull, highlighting its fundamental advantage as a protein source. More importantly, both its digestible crude protein and metabolizable protein levels rank second, far exceeding those of other raw materials except rapeseed pod hull. This indicates that the protein in peanut hulls not only has a relatively high total quantity but also exhibits superior efficiency in actual digestion, absorption, and conversion into usable forms by livestock, offering greater practical value for effective nitrogen supplementation. Regarding fiber composition and mineral characteristics, peanut husk records the highest levels of neutral detergent fiber and acid detergent fiber among the five raw materials. This property enables it to more effectively stimulate rumen motility and promote microbial fermentation when used as roughage for ruminants, thereby maintaining homeostasis in the rumen environment. In terms of minerals, peanut husk contains significantly higher calcium levels than rice hull, sunflower seed hull, and cottonseed hull (only lower than rapeseed pod hull), providing adequate calcium resources critical for skeletal development and physiological metabolism in livestock. However, its phosphorus and fat contents are relatively low, resulting in a somewhat limited contribution to energy supply through lipid sources. During application, attention should be paid to pairing it with high‐phosphorus ingredients to balance the calcium‐to‐phosphorus ratio. Overall, peanut hulls exhibit outstanding performance in dry matter stability, organic material basis, protein utilization efficiency, and calcium nutrition supply. Their high fiber characteristics meet the nutritional needs of ruminants. Although there is a limitation of low phosphorus content, their comprehensive nutritional value offers significant advantages among shell‐derived raw materials, and their application potential as feed ingredients deserves further exploration.

**TABLE 1:** Comparison of the nutritional values of peanut hulls and other agricultural by‐products.

Items^a^	Peanut hulls	Rice hulls	Sunflower seed hulls	Rapeseed pod hulls	Cotton boll hulls
Dry matter	94.00	93.43	88.00	92.32	91.12
Organic matter	95.78	92.86	97.30	83.40	90.40
Crude protein	5.08	4.73	3.60	9.57	3.95
Crude fat	0.90	1.24	1.98	3.22	1.75
Neutral detergent fiber	73.25	71.89	65.7	48.24	61.68
Acid detergent fiber	59.31	36.63	30.40	26.35	50.91
Crude ash	4.22	7.14	2.70	16.6	9.60
Calcium	0.80	0.16	0.29	1.37	0.55
Phosphorus	0.05	0.06	0.03	0.21	0.09
Digestible crude protein	1.89	1.57	0.56	5.91	0.88
Metabolizable protein	1.26	1.08	0.48	3.65	0.66

^a^ The above data is quoted from Agricultural Industry Standards of China (Ministry of 2021).

Although peanut hulls contain various nutrients, they present certain challenges in feed applications. From the perspectives of gas production characteristics and digestibility (Table 2), peanut hulls have a dry matter content of 96.11% and contain diverse nutrients and minerals. However, their insoluble fraction exhibits low fermentability, a slow gas production rate, and limited potential gas yield. In vitro dry matter and organic matter digestibility, along with cumulative gas production, remain low at all tested time points, resulting in an estimated metabolic energy value of 4.48 MJ/kg *DM* (Chumpawadee et al. 2007). These characteristics indicate that the high fiber and lignin content reduce microbial accessibility in the rumen, leading to low fermentation efficiency, suboptimal digestibility, and lower energy density compared to conventional feedstuffs. Using the nylon bag technique, the dry matter degradation rate (dt) of peanut hulls was evaluated at 38.3%–50.1%, classifying them as low‐quality forage (dt < 50% *DM*). The readily degradable fraction accounts for 26.2%, while the slow‐degradable fraction ranges from 29.2% to 49.35%, with a degradation rate of 0.156%–0.207%/h (Fall et al. 1998). Studies by Bizzuti et al. (2023) further characterize peanut hulls as having low crude protein (comparable to orange peel residue and sugarcane bagasse), high fiber content, minimal soluble carbohydrates, moderate methane production efficiency, and a slow fermentation rate (Liu et al. 1994). Multivariate analysis categorizes them as roughage via cluster analysis and principal component analysis. Despite these limitations, peanut hulls offer synergistic advantages in feed formulation. For instance, when the concentrate‐to‐roughage ratio is 40:60, increasing the proportion of peanut hulls relative to alfalfa hay enhances the associative effect of the diet. Peak 24‐h gas production and combined effect value occur at a 40% peanut hull inclusion level, with the optimal ratio being peanut hulls: alfalfa hay = 40:20 (He et al. 2020). Optimal synergies were also observed in blends of concentrated feed, peanut hulls, and alfalfa. At a concentrate‐to‐roughage ratio of 40:60 with 30% peanut hulls, and 30:70 with 10% peanut hulls, the mixtures demonstrated superior 48‐h gas production, dry matter digestibility, organic matter digestibility, and total volatile fatty acid concentrations. The most effective ratios were concentrate: peanut hulls: alfalfa = 40:30:30 and 30:10:60 (Yuan and Wan 2019). Additionally, a substrate mixture of corn straw and peanut hull powder at a 3:7 ratio maximized rumen microbial metabolic activity and growth proliferation, followed by a 4:6 ratio (Fu et al. 2014). In conclusion, despite their nutritional limitations, peanut hulls can contribute to livestock production through strategic combination with other feedstuffs. Research on their formulation provides valuable insights for utilizing unconventional feed resources in animal nutrition.

**TABLE 2:** Ruminal degradation rate and degradation parameters of different peanut shells.

Items	a (ml)^a^	b (ml)^b^	a + b (ml)^c^	c (%/h)^d^	IVDM 24 h^e^	GV24 (ml/0.5 g DM)^f^	References
Brahman‐Thai native crossbred steers	2.74	6.34	63.08	0.017	5.25	22.6	Chumpawadee et al. (2007)
Du × Han F1 castrated Rams	3.31	20.48	—	0.017	9.85	—	Chen et al. (2014)
30 kg Small Tail Han Sheep	2.71	44.25	46.96	0.059	—	36.5	Jiu et al. (2019)
32 kg Small Tail Han Sheep	9.0	50.95	59.96	0.10	—	34	He et al. (2020)
Steppe red cattle	6.14	45.57	51.74	0.03	22.55	—	Zheng et al. (2020)

^a^ a was the gas production of the fast fermentation part.

^b^ b was the gas production of the slow fermentation part.

^c^ a + b was potential gas production.

^d^ c was the gas production rate.

^e^ IVDM 24% was in vitro dry matter degradability 24 h.

^f^ GV24 was gas volume at 24 h.

### 2.2 Bioactive Components of Peanut Shells

In the current context where the utilization of agricultural waste resources has garnered significant attention, peanut hulls—a long‐overlooked by‐product of peanut processing—have emerged as a focal point in scientific research owing to their diverse functional constituents. As a valuable biological resource, peanut hulls have been extensively studied for their bioactive compounds. They comprise substantial amounts of hemicellulose, cellulose, and lignin, which confer unique physical properties and structural integrity (Ministryof 2021), alongside essential nutrients such as proteins, fats, carbohydrates, sugars, and minerals (Chen et al. 2014). Notably, peanut hulls are enriched with bioactive molecules, including polyphenols, flavonoids, carotenoids, and luteolin. These components are not only safe but also exhibit potent biological activities, including antioxidant and antibacterial effects (Jiu et al. 2019; Zheng et al. 2020; Prabhakar et al. 2015). Their antioxidant capacity effectively scavenges free radicals, mitigates oxidative stress, and offers significant health benefits (Adhikari et al. 2019). Furthermore, these properties deter insect pest infestations (Gao 2024), drawing interest from chemists and nutritionists (Zhang et al. 2013; Rosales et al. 2014). The high content of phenolic and flavonoid compounds underpins the pronounced antioxidant potential of peanut hulls, enabling them to reduce oxidative damage significantly. In conclusion, the multifaceted functional components of peanut hulls warrant deeper investigation for their potential applications across various fields.

In recent years, peanut shells—an abundant biological resource—have garnered extensive attention owing to their rich repertoire of bioactive and functional compounds (Table 3). A study by Adhikari et al. (2019) revealed that six peanut shell varieties in South Korea displayed DPPH radical scavenging rates of 61.9%–87.6% and ABTS scavenging rates of 39.6%–58.1%, with total phenol levels spanning 428.1–739.8 μg gallic acid equivalents (GAE)/g and flavonoid contents from 142.6–568.0 μg quercetin equivalents (QE)/g. These findings robustly indicate the significant antioxidant capacity of peanut shells. Similarly, Sodari and Gabiash's peanut shells in Sudan exhibited remarkable antioxidant activity, with total phenol and flavonoid contents reaching 66.3–85.3 mg GAE/g and 24.9–28.3 mg catechin equivalents (CE)/g, respectively, alongside DPPH scavenging activity of 80.9%–82.5% (Yu et al. 2014). These advances not only corroborate the antioxidant potential of peanut shells but also furnish a scientific foundation for their application in antioxidant‐driven industries. Significantly, major flavonoids isolated from peanut shells‐including 5,7‐dihydroxychromone, eriodictyol, and luteolin (Daigle et al. 1988), exhibit potential in regulating blood glucose levels and demonstrate promise in anti‐cancer activities (Jung et al. 2014; Laura et al. 2017). Among these, luteolin (LUT), a principal bioactive constituent, exhibits particularly pronounced biological activity. Under defined processing conditions (75% ethanol as solvent, solid‐to‐liquid ratio of 1:20 w/v, extraction temperature of 80°C, and duration of 2.0 h), LUT extraction yield reached 0.32 mg/g with a purity of 67.86% (Wang et al. 2021). Critically, animal studies reveal that the bioavailability of LUT extracted from peanut shells surpasses that of pure LUT (Zhou et al. 2008). In cellular lipid metabolism research, Liu et al. (Liu, Wang, and Zhang 2022) observed that high concentrations of peanut shell extract significantly reduced mRNA levels of C/EBP, PPAR, and SREBP1‐c while elevating pACC and pAMPK protein levels, thereby suppressing adipogenesis. This observation elucidates the mechanism underlying LUT's role in lipid regulation and supports its therapeutic potential for obesity and metabolic disorders. Beyond antioxidant activity, LUT exhibits notable anti‐inflammatory, anti‐allergic, and anti‐diabetic properties (Zang et al. 2016; De Stefano et al. 2021; Gaihre et al. 2022; Conti et al. 2021), broadening its application prospects in pharmaceuticals and nutraceuticals. However, LUT content varies markedly across cultivars; intermediate, multi‐grain, and pearl bean types exhibit higher concentrations (Ding et al. 2005). For instance, Spanish, Valencia, Runner, and Virginia varieties contain 3.16, 2.47, 0.95, and 0.75 mg/g LUT, respectively, with corresponding total phenolic contents of 10.2, 10.1, 6.6, and 4.2 mg/g (Yen and Duh 1995). This variability underscores the necessity of considering genetic factors during resource selection. Peanut shells additionally harbor diverse bioactive molecules such as diosmetin, ferulic acid, β‐sitosterol, and daucosterol (Zuo et al. 2015), collectively contributing to antioxidant, antibacterial, and anti‐aging effects (Choi et al. 2014). Innovations in extraction techniques further enhance yield: ultrasonic‐assisted ethanol extraction achieved β‐carotene yields of 5.33 g β‐carotene equivalents (TE)/100 g (Imran et al. 2022), while ultra‐high pressure extraction yielded 71.3 mg GAE/g polyphenols under optimized conditions (Yu et al. 2016). These advancements expand peanut shell applications in food additives, cosmetics, and functional foods. In conclusion, peanut shells represent a versatile resource with substantial potential for sustainable utilization. Future research should prioritize optimizing extraction technologies and exploring novel applications of their bioactive constituents to maximize industrial and ecological benefits.

**TABLE 3:** The active ingredients and physiological functions of peanut shells.

Item	Compound name	Content	References	Biological activity	References
1	Epigallocatechin	70.34 μg/g^−1^	(Ambrogina et al. 2020)	Antioxidant, anti‐inflammatory, anti‐tumor, protect the cardiovascular system	Auclair et al. (2009); Haza and Morales (2011); Mokra et al. (2022); Song et al. (2024); Talib et al. (2024); Zhang et al. (2024); Zheng et al. (2025)
2	Catechin	108 μg/g^−1^			
3	Epicatechin	100.09 μg/g^−1^			
4	P‐coumaric acid	21.73 μg/g^−1^		Immunoregulation, antioxidant	Zheng et al. (2025); Roychoudhury et al. (2021)
5	Ferulic acid	6.53 μg/g^−1^		Antibacterial and anti‐inflammatory	Liu, Shi, et al. (2022); Ordoñez et al. (2022)
6	Luteolin	1367.19 μg/g^−1^		Antioxidant, antibacterial, anti‐inflammatory, regulation of microbial flora	Iida et al. (2024)
7	Lunamarine	0.69 μg/mL	Nnabugwu and Uchenna's (2019)	Vasodilation	Agboola et al. (2024)
8	Kaempferol	2.44 μg/mL		Antioxidant, anti‐apoptosis, anti‐virus	Zhou et al. (2023); Jin et al. (2023)
9	β‐carotene	5.33 g TE/100 g	Imran et al. (2022)	Antioxidant and anti‐inflammatory	Gezer et al. (2024); Habtegebriel et al. (2024)

### 2.3 Anti‐Nutritional Factors of Peanut Shells

Although peanut shells possess high nutritional value, intrinsic characteristics impede their widespread utilization in animal feed formulations. Bitterness and high‐level protein‐binding constituents adversely influence animal feeding behavior, consequently diminishing feed intake (Dean 2020). The primary anti‐nutritional factors encompass phenols, phytic acid, lectins, and mycotoxins. Notably, the concentrations of these compounds vary significantly based on raw material origin and processing methodologies. Existing research remains limited concerning the nutritional profile of peanut shells, particularly regarding anti‐nutritional factor contents. Nevertheless, prior investigations report that peanut shells contain oxalic acid (220 mg/100 g), phytic acid (362.1 mg/100 g), and glycosides (1.60 mg/100 g), with trypsin inhibitor activity measured at 25 trypsin inhibitor units (TUI)/mg (Abdulrazak et al. 2014). Sim et al. (Sim et al. 2012) identified considerable genotypic variations in peanut shell composition, observing Menglembu cultivars with 159 mg/100 g phytic acid and 10.8 mg/100 g saponins, contrasted with Shandong peanut shells containing 108 mg/100 g phytic acid and 23.4 mg/100 g saponins. Phytate chelates minerals such as calcium and iron, reducing their bioavailability, while oxalic acid precipitates calcium oxalate crystals, potentially causing renal tubular obstruction (Sun 2020). However, all detected levels remain below established toxicity thresholds: cyanogens and phytate (50–60 mg/kg), oxalic acid and trypsin inhibitors (2–5 g/kg) (Onwuka 2005). With appropriate processing, peanut shells can serve as viable animal feed ingredients. Tannin content in peanut shells exhibits substantial variability influenced by cultivar, environmental conditions, maturity stage, and processing techniques. For instance, Hill et al. (1987) reported tannin levels in peanut skins ranging from 16% to 24%, whereas Abid et al. (2024) measured condensed tannins at 330 μg catechin equivalents (CE)/g. Nnabugwu and Uchenna's (2019) study found that peanut shell oil also has a certain amount of anti‐nutritional factors, including phytic acid content of 53.81 mg/mL, oxalic acid 39.47 mg/mL, and tannin 17.03 mg/mL. In conclusion, peanut shells harbor diverse anti‐nutritional factors with highly variable concentrations, posing significant challenges for animal feed applications. Effective mitigation strategies‐such as physical treatment, enzyme supplementation, or solvent extraction essential to reduce these compounds and enhance feed safety. Future research should prioritize optimizing processing protocols to minimize anti‐nutritional impacts while preserving nutritional quality, thereby facilitating sustainable utilization of peanut shells in livestock production systems.

## 3 Processing and Treatment Technologies of Peanut Hulls

In the context of today's pursuit of sustainable agricultural development, improving the comprehensive utilization of peanut shells and reducing environmental pollution have become key issues. Although the crude protein content of peanut shells is lower than that of traditional protein feeds such as soybean meal, they retain value as a roughage supplement or functional additive. However, high cellulose content limits their direct utilization by monogastric animals. Nevertheless, physical or chemical pretreatments‐including crushing and fermentation, significantly enhance digestibility and expand their application potential in livestock production.

Firstly, physical treatments have yielded remarkable results. Ground peanut shell powder processed into dust‐free particles minimizes feed dust, thereby improving intake and digestibility in feed formulations (Mandala et al. 2023). This improvement likely results from physical disruption of the crystalline structure, reduced particle size, and decreased crystallinity (Feng et al. 2022). Carbonaceous biomass produced by high‐temperature pyrolysis of peanut shells exhibits strong adsorption capacity, enhancing nutritional value as a specialized feed additive (Hashem et al. 2024). Emerging technologies such as anaerobic fermentation, ultrasonic treatment, and organic solvent extraction further optimize peanut shell utilization in animal feed (Erickson et al. 2014; Oliva et al. 2023). Secondly, chemical treatments play a critical role. Alkali treatment studies demonstrate that increasing NaOH concentrations linearly reduce neutral detergent fiber while boosting in vitro dry matter digestibility (Chandra et al. 1985). In contrast, calcium oxide treatment yielded no significant improvements in ruminal in situ degradability; instead, in vitro organic matter digestibility followed a biphasic pattern (initial increase followed by decline), with calcium oxide reducing digestibility under specific conditions (Ciriaco et al. 2016). The initial digestibility rise likely stems from alkali‐induced disruption of hydrogen and glycosidic bonds within the lignocellulose matrix, loosening its dense structure and improving fiber accessibility.

Fermentation stands as a core strategy for valorizing peanut shells. Probiotic fermentation with *EM‐4* enhances nutritional profiles and digestibility while suppressing pathogen proliferation, reducing disease risks (Armayanti et al. 2021). Semi‐solid fermentation with 2% liquid composite probiotics increases crude protein by 6.3%, nitrogen‐free extracts by 3.7%, and reduces crude fiber by 12.5% (Jiang et al. 2009). Treatments combining *Trichoderma viride*, urea, and cellulase similarly elevate crude protein, dry matter, and organic matter digestibility, while lowering crude fiber content (Abo‐Donia et al. 2014) and increasing non‐fibrous carbohydrate and soluble organic matter concentrations (Abid et al. 2024). Supplementation with nutritional additives during fermentation further elevates organic acids, crude protein, and water‐soluble carbohydrate levels, concomitantly inhibiting harmful microbial growth (Pan et al. 2019). Practical applications often involve co‐silencing peanut shells with complementary substrates. Ensiling with soybean milk residue at a 78:22 ratio optimizes fermentation efficiency and rumen fermentation characteristics (Yang 2005), while incorporating peanut shells into seafood waste silages improves feed odor and reduces *Escherichia coli* and fecal coliform counts (Samuels et al. 1992). These practices demonstrate that functional properties can be tailored through raw material ratio optimization and fermentation parameter control. In conclusion, while fermentation enhances peanut shell quality, its efficacy depends on dynamic interactions among microbial consortia, additive compositions, and substrate ratios. Standardization of critical parameters‐including microbial strain specificity, additive concentrations, and co‐substrate ratios‐is essential for practical implementation. Future research should elucidate microbial community synergism and processing condition interactions, validate long‐term effects across diverse husbandry systems, and establish evidence‐based directional fermentation strategies for peanut shells.

## 4 APPLICATION OF PEANUT SHELL IN ANIMAL HUSBANDRY PRODUCTION

### 4.1 Application in Poultry Production

**FIGURE 3:**
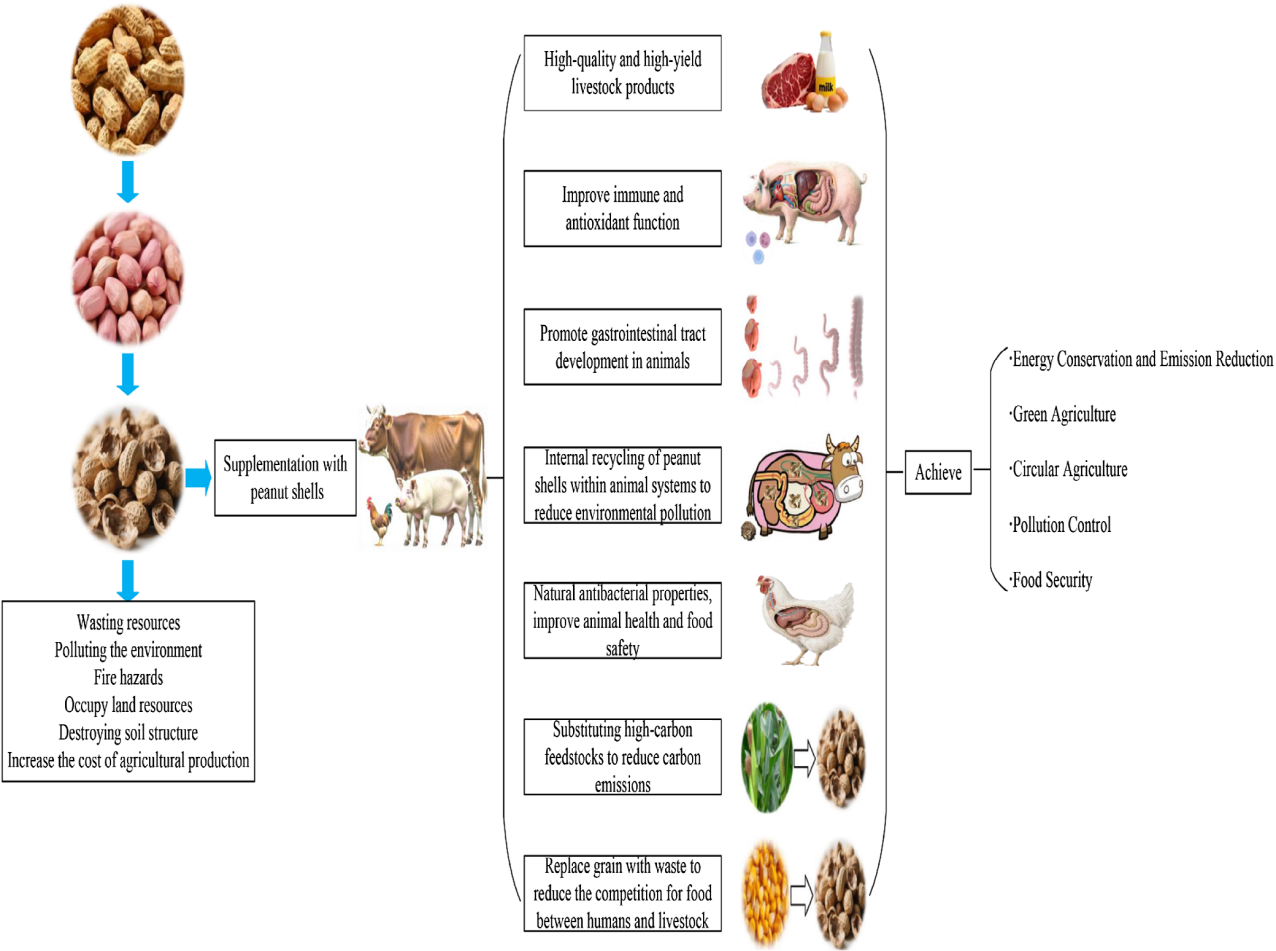
Application potential of peanut shells in poultry and livestock production.

In the research realm of comprehensive agricultural resource utilization, peanut shells, an abundant agro‐byproduct, garner increasing scientific interest, particularly regarding the putative health and immunomodulatory effects of their bioactive constituents (Figure 3). Plant‐derived natural bioactive compounds and trace mineral elements hold promise as novel feed additives to supplant antibiotics, thereby efficaciously enhancing the health status of livestock and poultry (Saeed, Hassan, et al. 2025; Saeed, Khalaifah, et al. 2025; Anggriawan et al. 2024; Yasmeen et al. 2023). Akiba and Matsumoto (1980) demonstrated that peanut hulls modulate hepatic and plasma lipid profiles in growing chicks, elevate plasma protein concentrations, augment bile acid excretion, and mitigate liver injury. Furthermore, fermented peanut hulls have been shown to elicit beneficial effects when incorporated into feed formulations. In broiler production, graded supplementation of fermented peanut hull powder improved body weight gain and feed conversion ratio without affecting voluntary feed intake, with optimal performance and economic returns achieved at a 5% inclusion rate (Armayanti et al. 2021). Subsequent investigations confirmed that peanut hull extracts confer substantive benefits to poultry productivity and well‐being. Supplementation with peanut hull extracts at 400, 700, or 1000 g/ton led to reduced oxidative stress biomarkers, enhanced antioxidant enzyme activities, increased egg production rates, and improved feed efficiency in 49‐week‐old Jingfen layer hens, with optimal outcomes achieved at inclusion rates of 700 and 1000 g/ton (Yang et al. 2018). Additionally, Han et al. (2016) reported that dietary incorporation of 0.05% or 1% peanut hull powder significantly augmented body weight, compensatory growth capacity, and feed efficiency in Ross 308 broilers, while improving meat cooking loss and crude protein retention. These findings collectively underscore the multifaceted utility of peanut hulls in poultry nutrition. Nevertheless, the practical efficacy of peanut hulls in poultry systems is multifactorially modulated by processing protocols, dosage compatibility with avian physiological thresholds, developmental phases, and production objectives. Future research endeavors should elucidate the dose–response relationships between specific peanut hull components (e.g., dietary fiber, polyphenols, fermentation metabolites) and avian physiological outcomes, while optimizing processing methodologies and inclusion strategies. Through precision formulation, tailored approaches can address the distinct nutritional and physiological requirements of diverse poultry genotypes (broilers, layers, breeders) across developmental phases (chick, grower, reproductive stages), thereby enabling evidence‐based, high‐efficiency utilization of peanut hulls in sustainable poultry production systems.

### 4.2 Application in Monogastric Animal Production

In the field of monogastric animal farming, research on the application of peanut shells has been continuously deepened, with their effects on different species exhibiting diversified characteristics (Table 4). Many studies have focused on the role of peanut shells in finishing pigs, providing valuable references for optimizing swine feed formulations. Liu et al. (2012) found that replacing 10% corn with peanut shells had no adverse effect on feed palatability, improved meat quality, reduced the feed‐to‐meat ratio, and increased economic benefits. Zhang et al. (2015) similarly demonstrated in their study on fermented peanut shells in finishing pigs that adding graded levels (10%, 20%, 30%) to the basal diet did not compromise production performance, slaughter traits, or pork quality, while effectively reducing diarrhea incidence. Additionally, Jiang et al. (2009) showed that replacing 15% of complete feed with 2% probiotic‐fermented peanut shell powder had no significant impact on weight gain or feed‐to‐gain ratio but reduced diarrhea incidence and fecal, highlighting its potential as a partial feed replacement and antidiarrheal agent. This shows that peanut shells can dilute dietary energy, prolong digestion time, reduce the metabolic risk of high‐energy and high‐protein feed, improve fecal traits, reduce constipation, and are especially suitable for animal groups with limited exercise. However, studies in piglets reveal contrasting outcomes. In vitro analyses indicated that peanut shells had the lowest in vitro ileal digestibility and in vitro total digestive tract digestibility (Zhang et al. 2022), with multi‐enzyme supplementation further reducing in vitro digestibility (Recharla et al. 2019). Consistent with this, Kornegay et al. (1995) observed in 25‐day‐old weaned piglets that an 8% peanut shell inclusion increased the feed‐to‐gain ratio and decreased average daily gain. Lindemann et al. (1986) reported analogous findings in 72‐kg hybrid sows: while 7.5%, 15%, and 30% peanut shell additions improved certain mineral metabolism parameters, they reduced nitrogen, energy, and fiber digestibility, with daily gain declining linearly as inclusion levels increased. Comparable results were documented in 2‐month‐old New Zealand rabbits (Chao and Li 2008), where optimal production performance and gastrointestinal development occurred at 70 g/kg peanut shell inclusion (accompanied by increased cecal acetic acid concentration and fibronolytic activity), but performance deteriorated linearly at higher doses. In conclusion, peanut shells represent a low‐cost dietary fiber source but contain high lignin content and antinutritional factors. Their efficacy in monogastric animals is constrained by physiological stage, processing method, and inclusion level. Moderate processing can modify functional components; however, unprocessed or excessive inclusion may impair performance due to antinutritional properties. A successful application requires precise alignment with digestive capacities, technological disruption of digestive barriers, and strict control of inclusion rates. Future research should establish dynamic inclusion guidelines tailored to animal physiological stages, optimize fermentation parameters to enhance fiber conversion efficiency, elucidate fiber‐microbiota‐host interaction mechanisms, transcend empirical substitution limits, and achieve the scientific transformation of peanut shells from waste to functional feed resources.

**TABLE 4:** Application of peanut shells in livestock and poultry production.

Classification	Breed	Adding amount	Effect	References
Poultry	49‐week‐old Jingfen laying hens	400, 700, and 1000 g/t	Improve egg production, laying rate, and feed intake, enhance the activity of antioxidant enzymes, and reduce oxidative stress levels	Yang et al. (2018)
	Broiler chicken; Ross 308	0.05% and 0.1%	Increase total weight gain, compensate for growth rate and feed conversion efficiency, improve cooking loss, crude protein content, and antioxidant activity	Han et al. (2016)
	Day old chicken	5%, 10% and 15% fermented peanut shell meal	Improve the growth performance, reduce the ratio of material to weight	Armayanti et al. (2021)
Monogastric animal	70 and 58 kg growing finishing pigs	10%	Improve meat quality, reduce the ratio of feed to meat, and improve economic efficiency	Liu et al. (2012)
	50 kg Duroc × Landrace × Yorkshire crossbred fattening pigs	10%, 20% and 30% Fermented peanut shell	Reduce the rate of diarrhea, improve meat quality, and improve economic efficiency	Zhang et al. (2015)
	35–45 kg three‐way crossbred pigs	2% fermented peanut shell powder	Reduce diarrhea rates and fecal odor	Jiang et al. (2009)
	28‐day‐old weaned piglets	8% and 16%	Reduced feed efficiency, average daily gain, and fiber digestibility	Kornegay et al. (1995)
	72 kg crossbred sows	7.5%, 15% and 30% peanut shells	Reduce nutrient digestibility and daily weight gain, increase fecal production, and reduce urine volume	Lindemann et al. (1986)
	2‐month‐old New Zealand rabbits	30, 70, 105, and 195 g/kg	Improve daily weight gain, promote gastrointestinal development, improve intestinal fermentation, and fiber decomposition	Chao and Li (2008)
Ruminant	12 kg male Hu sheep	30%, 40% and 50%	Increase daily gain, reduce feed‐to‐gain ratio	Chen et al. (2022)
	15 kg male Hu sheep	20% and 40% fermented peanut shells	Increase daily gain, reduce feed‐to‐gain ratio, improve immunity, and liver and kidney function	Zhou et al. (2022)
	64 kg Ossimi rams	Peanut shell fungal treatment and urea treatment	Improve feed intake, nutrient digestibility, and rumen fermentation function	Abo‐Donia et al. (2014)
	35 kg desert ram	20%	Improve digestibility and rumen fermentation, increase serum glucose	Maglad et al. (1986)
	35 kg crossbred wether lambs	16.8%	Increase the concentration of acetic acid, propionic acid, butyric acid, and isovaleric acid in the rumen	McCarthy et al. (1990)
	25 kg male sheep	18%	Improve dry matter digestibility and reduce fiber digestibility	Fall et al. (1998)
	16.5 kg Nellore ram lambs	40%	Improve digestibility, water intake, urine volume, and urine pH	Chandra et al. (1985)
	13 kg male lamb	20%	Increasing feed intake, daily gain, dry matter, and organic matter digestibility, and reducing to gain‐to‐feed ratio	Purbowati et al. (2021)
	Adult Yanbian Yellow Cattle	Fermented peanut shells and unfermented peanut shells (73% and 37%)	Promote rumen fermentation and reduce in vitro fermentation gas production	Ma et al. (2016)
	South African native cattle	700, 1050, 1400 and 1750 g/kg	Improve metabolic function, increase blood urea and lipase content	Mokolopi et al. (2021)
	650 kg Holstein cows	Chounfakhi variety peanut shells	Accelerate fermentation rate, improve fiber digestibility, and increase short‐chain fatty acid production	Abid et al. (2024)
	267 kg *Bos indicus* steers	30% peanut hull fiber pellet	Feed intake, digestibility, and rumen microbial protein synthesis were not affected	Pan et al. (2023)
	233 and 301 kg bulls	45% peanut hulls	Reduces daily weight gain and feed intake	Kennedy and Rankins (2008)

### 4.3 Application in Ruminant Production

As an agricultural by‐product, peanut shells exhibit low digestibility and limited practical utility due to their high crude fiber content and low crude protein concentration. Nevertheless, substantial evidence indicates that judicious processing and formulation enable peanut shells to exert beneficial effects in ruminant production systems (Table 4). In a growth trial with 12‐kg male Hu sheep, substituting 30%, 40%, and 50% of peanut seedling meal with fermented peanut hulls enhanced average daily gain (ADG) by 8.04%, 12.58%, and 5.7%, respectively (Chen et al. 2022). Subsequent studies in 15‐kg Hu sheep confirmed these findings: replacing 20% and 40% of peanut seedling meal with fermented peanut hulls elevated ADG and serum immunoglobulin concentrations while reducing feed conversion ratio (FCR) (Zhou et al. 2022). These results suggest that the strategic inclusion of fermented peanut hulls can promote growth and immune function in Hu sheep, potentially due to bioactive compounds such as polyphenols, flavonoids, carotene, and luteolin, known for their antioxidant and antibacterial properties that scavenge free radicals and alleviate oxidative stress (Zhang et al. 2013; Rosales et al. 2014; Yu et al. 2014).

Responses to peanut hull inclusion levels and processing methodologies exhibit variable impacts on nutritional metabolism and rumen fermentation parameters across studies. For instance, replacing 20% elephant grass with peanut hulls in 13‐kg ram lambs improved feed intake, digestibility, and ADG while lowering FCR (Purbowati et al. 2021). Conversely, supplementing 25‐kg male sheep with 18% peanut hulls elevated dry matter digestibility but reduced acid detergent fiber digestibility and other nutrient utilization metrics (Fall et al. 1998), likely reflecting the interplay between the inherent high neutral detergent fiber and lignin content of peanut hulls and the elevated concentrate proportion in the experimental diet. Studies by McCarthy et al. (1990) and Maglad et al. (1986) demonstrated that diets containing 16.8% and 20% peanut hulls for 35‐kg crossbred lambs and desert sheep slightly reduced fiber digestibility and nitrogen absorption but increased ruminal acetate concentrations and serum glucose levels. Similarly, in vitro experiments revealed that alkali treatment improved hemicellulose digestibility and water‐mineral metabolism (Chandra et al. 1985), while fungal or urea treatment enhanced feed intake, digestibility, and rumen function in Ossimi rams, accompanied by increased microbial populations (Abo‐Donia et al. 2014).

The potential of peanut shells as a ruminant feed resource has been explored through extensive in vitro and in vivo trials, yielding context‐dependent outcomes. In vitro fermentation studies show that fermented peanut shells accelerate rumen fermentation rates and enhance fiber and dry matter digestibility (Ma et al. 2016). Trials with Yanbian cattle indicated that unfermented peanut shells minimally affected rumen pH and ammonia nitrogen concentrations, whereas fermented shells promoted microbial activity and reduced methane emissions‐with high‐concentrate fermented shells being particularly suitable for rumen microbiota (Abid et al. 2024). In vivo evaluations in South African indigenous cattle revealed dose‐dependent adverse effects: blood metabolites declined progressively with increasing peanut shell inclusion (700, 1050, 1400, and 1750 g/kg diet), with significant metabolic disruptions observed at 1050 g/kg (Mokolopi et al. 2021). Under tropical grazing conditions, however, a 30% peanut shell‐fiber supplement had no significant impact on feed intake, digestibility, or microbial protein synthesis in Indian bulls (Pan et al. 2023). In hybrid beef cattle, sole peanut shell supplementation reduced ADG and feed intake without affecting digestibility, but co‐inclusion of 8% cottonseed meal improved intake and crude protein digestibility (Kennedy and Rankins 2008). In conclusion, the efficacy of peanut shells as a roughage source for ruminants is modulated by processing techniques, inclusion levels, animal species, and management practices. While abundant and cost‐effective, their nutritional limitations necessitate balanced formulation with complementary feedstuffs and protein supplements to optimize nutrient utilization. Appropriately processed peanut shells provide fermentable fiber for rumen regulation, release bioactive antioxidants to bolster immunity, and serve as a low‐cost carrier for co‐products like cottonseed meal. In meat sheep systems, inclusion levels below 20% yield optimal economic returns. Practical implementation faces challenges, including scalable fermentation costs and knowledge gaps regarding bioactive compound mechanisms. Future research should prioritize elucidating these aspects to develop evidence‐based protocols for maximizing peanut shell value in ruminant agriculture.

## 5 Conclusion

Peanut shells exhibit significant potential in animal nutrition, contributing to reduced feeding costs, enhanced animal health, and environmental sustainability. Characterized by high fiber and low fat content, they hold development value despite having lower crude protein levels than soybean meal. Applications in poultry, monogastric animals, ruminants, and nutrition have been documented, though careful consideration of inclusion rates and formula compatibility is essential. Future research should prioritize in‐depth studies on processing methods and their impacts on nutritional components, alongside increased R&D investment to optimize feed formulation through meticulous experimentation. This aims to address feed resource shortages and environmental challenges. In conclusion, ongoing exploration of advanced processing technologies and application strategies for peanut shells is crucial to fully unleash their value in animal nutrition, offering innovative solutions to current feed resource scarcity and environmental issues.

## Author Contributions

**Dian Wang, Yingli Li:** conceptualization. **Mengen Zhang, Hongsheng Du:** data curation. **Mengen Zhang, Guodong Li:** formal analysis. **Mengen Zhang, Hongsheng Du:** methodology. **Dian Wang:** software. **Mengen Zhang, Hongsheng Du:** validation. **Mengen Zhang, Hongsheng Du, Rubing Lan:** investigation. **Mengen Zhang, Shiqin Wang, Rubing Lan, Dian Wang, Yingli Li:** writing – original draft. **Shiqin Wang, Dian Wang:** writing – review and editing.

## Ethics Statement

The authors have nothing to report.

## Conflicts of Interest

The authors declare no conflicts of interest.

## Data Availability

Upon reasonable request, the datasets of this study can be made available from the corresponding author.
